# Gestational Diabetes and Health Behaviors Among Women: National Health and Nutrition Examination Survey, 2007–2014

**DOI:** 10.5888/pcd15.180094

**Published:** 2018-10-25

**Authors:** Fei Gao, Huabin Luo, Katherine Jones, Wanda Nicholson, Ronny A. Bell

**Affiliations:** 1 Department of Public Health, Brody School of Medicine, East Carolina University, Greenville, North Carolina; 2 Center for Women’s Health Research, Department of Obstetrics and Gynecology and the Center for Health Promotion and Disease Prevention, University of North Carolina, Chapel Hill

## Abstract

**Introduction:**

Women with gestational diabetes are at 7 times greater risk of developing type 2 diabetes than are women without gestational diabetes. The objectives of this study were to examine recent changes in the prevalence of gestational diabetes mellitus among women of reproductive age in the United States and assess the prevalence of factors associated with participating in healthy lifestyle behaviors.

**Methods:**

Data were from 4 waves of the National Health and Nutrition Examination Survey (2007–2014). Gestational diabetes was identified by participants’ response to whether they were ever told by a health care professional that they had diabetes during pregnancy. The health behaviors were participation in physical activity, healthy dietary patterns (intake of cholesterol, sodium, and fiber within recommended guidelines), and smoking. The analytical sample included 3,034 women aged 20 to 44 years. Multivariate logistic regression was used to assess the association between gestational diabetes and health behaviors.

**Results:**

The overall prevalence of gestational diabetes was 8.9% (95% confidence interval [CI], 7.6%–10.4%) during 2007–2014. The prevalence increased from 8.4% in 2007–2008 to 10.4% in 2013–2014, an increase of 24%, but the change was not significant (*P *= .28). The proportions of women meeting recommended guidelines for the health behaviors did not change significantly. We found no significant difference in practicing healthy behaviors between women with gestational diabetes and women without gestational diabetes.

**Conclusion:**

The prevalence of gestational diabetes increased slightly in recent years, and women with the condition were generally not meeting guidelines for healthy behaviors. Coordinated interventions are needed to promote healthy lifestyle behaviors among women with gestational diabetes because they are at increased risk for diabetes.

## Introduction

Gestational diabetes mellitus (hereinafter, gestational diabetes) is defined as any glucose intolerance diagnosed during pregnancy (1). An estimated 1% to 14% of pregnancies are affected by gestational diabetes in the United States (2,3). Women with gestational diabetes are at 7 times greater risk of developing type 2 diabetes than are women without gestational diabetes (4). Although most women with gestational diabetes return to normal glucose tolerance after delivery, as many as 10% to 50% can develop type 2 diabetes within 5 years (4). Yet, progression to type 2 diabetes can be prevented by adopting and maintaining a healthy weight through adopting healthy lifestyle behaviors (4,5).

Several studies have examined health behaviors among women with gestational diabetes. One analysis of data from the 2006 Behavioral Risk Factor Surveillance System found approximately 3% of women aged 18 to 44 had gestational diabetes, and levels of physical activity, fruit and vegetable consumption, or smoking did not differ significantly between women with gestational diabetes and women without gestational diabetes (5). Another study using data from the 2007–2010 National Health and Nutrition Examination Survey (NHANES) found that 7.7% of women aged 20 to 44 had gestational diabetes and that dietary quality among these women was lower than that among women without a history of gestational diabetes (6).

Information on trends in the prevalence of gestational diabetes is limited, and most of this information is based on hospital delivery data (7,8). Recent changes in health behaviors among women with a history of gestational diabetes in the United States have not been examined. Such information is needed to inform efforts to promote healthy lifestyles in this population. The objectives of our study were to 1) describe the prevalence and recent changes in the prevalence of gestational diabetes in the United States, 2) describe and compare changes in practicing healthy behaviors among women with and without gestational diabetes, and 3) assess the relationship between a diagnosis of gestational diabetes and practicing healthy behaviors. 

## Methods

We collected data for this analysis from 4 waves of NHANES: 2007–2008, 2009–2010, 2011–2012, and 2013–2014 (9). At the time of our analysis, in 2018, the most recent NHANES data available were from 2013–2014. NHANES consists of self-reported data collected from participants during an in-home interview and clinical examination data gathered in a mobile examination center. The survey provides estimates for health conditions and health behaviors that can be generalized to the entire US population. The question on gestational diabetes was first administered in 2007–2008. We collected measures of health behaviors from the questionnaires on physical activity, dietary recall, and smoking/cigarette use.

Our sample consisted of women aged 20 to 44 years. In the NHANES survey, women were asked, “During your pregnancy, were you ever told by a doctor or other health professional that you had diabetes, sugar diabetes or gestational diabetes?” As in previous research (6), women who responded yes to this question were classified as having gestational diabetes (n = 335); those who answered no or “borderline” were classified as not having gestational diabetes (n = 2,807). We excluded women who self-reported currently having diabetes (n = 108). Thus, the final study sample consisted of 3,034 women (287 with gestational diabetes and 2,747 without gestational diabetes): 802 women in 2007–2008, 833 women in 2009–2010, 625 women in 2011–2012, and 774 women in 2013–2014.

### Measures

**Physical activity.** In the Physical Activity Questionnaire, women were asked, “In a typical week, on how many days do you do vigorous-intensity sports, fitness or recreational activities?,” “How much time (minutes) is spent doing vigorous recreational activities?,” “In a typical week, on how many days do you do moderate-intensity sports, fitness or recreational activities?,” and “How much time (minutes) is spent doing moderate recreational activities?” To calculate the total minutes of physical activity, the number of minutes spent in vigorous-intensity physical activity was doubled (assuming that 1 minute of vigorous activity equals 2 minutes of moderate activity [10]) and added to the minutes of moderate-intensity physical activity (11). This variable was coded as binary: either meeting the guideline for physical activity (if ≥150 minutes per week) or not meeting the guideline (if <150 minutes per week).

**Dietary behaviors.** In the Dietary Recall Questionnaire, women were asked about their daily food consumption, and the amount of sodium, fiber, and cholesterol intake was estimated. According to the American Heart Association and the American Diabetes Association, a daily cholesterol intake of less than 300 mg, a daily fiber intake of more than 25 g, and a daily sodium intake of less than 1,500 mg meet the daily dietary guidelines (11). Daily intakes that met these guidelines were classified as meeting guidelines, and daily intakes that exceeded the guidelines (cholesterol or sodium) or did not reach the guideline (fiber) were classified as not meeting the guidelines. All 3 dietary behavior variables were coded as binary (yes or no) outcomes.

**Smoking behavior.** In the Smoking/Cigarette Questionnaire, women were asked, “Do you now smoke cigarettes?” and “Have you smoked at least 100 cigarettes in your entire life?” Women were classified as current smokers if they answered yes to both questions; otherwise, they were classified as noncurrent smokers (ie, former or never smokers).

On the basis of previous literature (5,12), we selected the following covariates: age, race/ethnicity (non-Hispanic white, non-Hispanic black, Mexican American, and other [other Hispanic people and other racial groups]), education (<high school graduate, high school graduate, ≥some college), ratio of family income to federal poverty level (<100%, 100%–199%, and ≥200%), marital status (married or living with partner, other), self-reported health (fair or poor; excellent, very good, or good), body mass index (normal/underweight [<25 kg/m^2^], overweight [25 to <30 kg/m^2^], and obese [≥30 kg/m^2^], and the number of times a delivery resulted in live birth.

### Statistical analysis

We calculated the overall prevalence of gestational diabetes during 2007–2014, and we examined differences in demographic characteristics between respondents with gestational diabetes and respondents without. For each survey period, we calculated the prevalence of gestational diabetes and the proportion of women who met the guidelines for healthy behaviors. We then assessed the changes (from 2007 through 2014) in gestational diabetes prevalence and proportions of women that met the guidelines for health behaviors, by regressing the prevalence rates and proportions of women who met the guidelines on time (ie, the survey period, coded as 1 for 2007–2008, 2 for 2009–2010, 3 for 2011–2012, and 4 for 2013–2014). Using the pooled data from 2007–2014, we ran multivariate logistic regression to assess the association between gestational diabetes and practicing the healthy behaviors, with the healthy behaviors being the outcome variables. We also tested the following interactions: gestational diabetes by year, gestational diabetes by race/ethnicity, gestational diabetes by income, and gestational diabetes by education. None of these interactions were significant. Thus, we did not include them in the final model. We used survey commands in SAS version 9.4 (SAS Institute Inc) in all analyses to account for the survey design of NHANES.

## Results

Women with a history of gestational diabetes were older (35.2 vs 33.7 y), more likely to be obese (48.4% vs 35.8%), more likely to be married (77.9% vs 70.9%), and more likely to report poor or fair health (23.1% vs 14.8%), and had more live births delivered (2.3 vs 2.1) than women without gestational diabetes (Table 1).

**Table 1:** Characteristics of a Sample (n = 3,034) of Women Aged 20–44 With and Without Gestational Diabetes, National Health and Nutrition Examination Survey, 2007–2014^a^

Characteristic	Women With Gestational Diabetes (n = 287)	Women Without Gestational Diabetes (n = 2,747)	*P* Value^b^
**Age, mean (95% CI),** **y**	35.2 (34.3–36.1)	33.7 (33.3–34.0)	.001
**Race/ethnicity**			
Non-Hispanic white	53.3 (44.5–62.0)	58.3 (53.2–63.3)	.18
Non-Hispanic black	13.7 (10.0–18.4)	15.2 (12.7–18.1)	
Mexican American	14.7 (9.8–21.4)	12.3 (10.0–15.1)	
Other	18.3 (14.1–23.4)	14.1 (12.0–16.6)	
**Married or living with partner**	77.9 (72.0–82.9)	70.9 (68.7–73.0)	.02
**Education level**			
<High school graduate	22.5 (17.2–28.7)	17.7 (15.8–19.7)	.20
High school graduate	17.2 (12.0–24.0)	20.6 (18.7–22.6)	
≥Some college	60.3 (52.2–67.9)	61.8 (58.9–64.5)	
**Ratio of family income to federal poverty level**			
<100%	21.6 (17.6–26.3)	23.3 (21.0–25.7)	.73
100%–199%	24.9 (19.9–30.8)	23.2 (21.7–24.8)	
≥200%	53.5 (46.7–60.0)	53.6 (50.6–56.5)	
**BMI**, **mean (95% CI)**	30.5 (29.5–31.6)	28.8 (28.4–29.1)	.02
**BMI category**			
Not overweight or obese (<25 kg/m^2^)	22.9 (18.6–27.8)	35.3 (32.9–37.8)	<.001
Overweight (25–30 kg/m^2^)	28.7 (23.0–35.3)	28.9 (27.1–30.8)	
Obese (30 kg/m^2^)	48.4 (41.3–55.5)	35.8 (33.8–37.8)	
**Health status fair or poor**	23.1 (18.1–29.0)	14.8 (13.2–16.7)	.001
**No. of live births delivered, mean (95% CI)**	2.3 (2.2–2.5)	2.1 (2.1–2.2)	.006

Abbreviations: BMI, body mass index, CI, confidence interval.

a All values are weighted percentage (95% CI) unless otherwise indicated. Means are weighted.

b Other Hispanic people and other racial groups.

The overall prevalence of gestational diabetes during 2007–2014 was 8.9% (95% confidence interval [CI], 7.6%–10.4%). The prevalence was 8.4% (95% CI, 6.2%–11.4%) in 2007–2008, 6.9% (95% CI, 4.7%–9.9%) in 2009–2010, 10.0% (95% CI, 7.8%–12.8%) in 2011–2012, and 10.4% (95% CI, 7.8%–13.9%) in 2013–2014. From 2007–2008 to 2013–2014, the prevalence of gestational diabetes increased 23.8%, but this increase was not significant (*P* = .28 according to linear regression model results).

**Figure 1:**
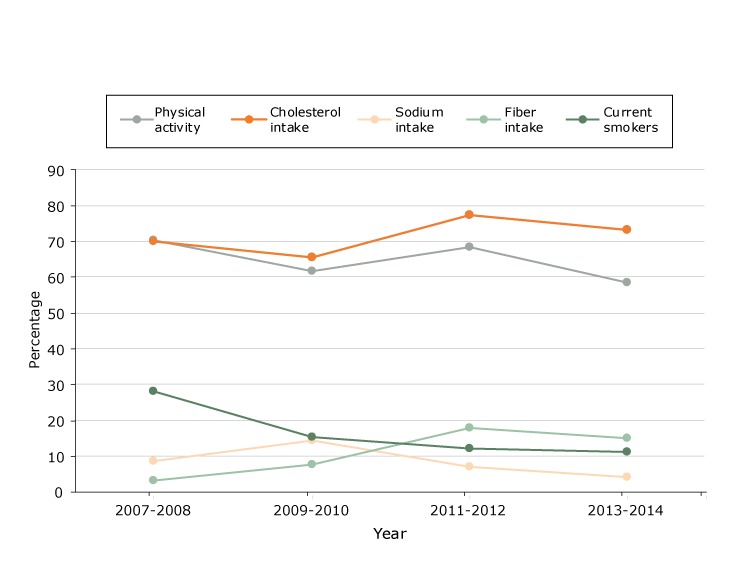
Proportions of women with gestational diabetes (n = 287) who met guidelines for health behaviors, National Health and Nutrition Examination Survey, 2007–2014. The estimates for fiber intake, sodium intake, and current smokers were not valid because the small sample size resulted in relative standard errors of more than 30%. **Table:** 

Changes in the proportion of women with a history of gestational diabetes who met the guidelines for health behaviors varied by behavior (Figure 1). For physical activity, the proportion decreased from 70.4% in 2007–2008 to 58.6% in 2013–2014, a decrease of 16.7% (70.38% − 58.60%/70.38%); for cholesterol intake, the proportion increased from 69.8% in 2007–2008 to 73.2% in 2013–2014, an increase of 4.8% (73.16% − 69.82%/69.82%). Overall, trends were flat, with no significant increases or decreases. The estimates for sodium intake, fiber intake, and current smokers were not valid because the small sample size resulted in relative standard errors of more than 30%.

Year	Physical Activity, %	Cholesterol Intake, %	Sodium Intake, %	Fiber Intake, %	Current Smokers, %
2007-2008	70.4	69.8	8.8	3.2	28.3
2009-2010	61.7	65.6	14.4	7.7	15.3
2011-2012	68.5	77.3	7.1	18.0	12.2
2013-2014	58.6	73.2	4.1	15.0	11.1

The pooled data for 2007–2014 showed that, among women with gestational diabetes, 64.2% (95% CI, 54.3%–73.0%) met the physical activity guideline, 71.8% (95% CI, 64.9%–77.8%) met the cholesterol intake guideline, 8.0% (95% CI, 4.9%–12.9%) met the sodium intake guideline, 11.3% (95% CI, 7.4%–17.0%) met the fiber intake guideline, and 16.5% (95% CI, 12.0%–22.2%) were current smokers.

**Figure 2:**
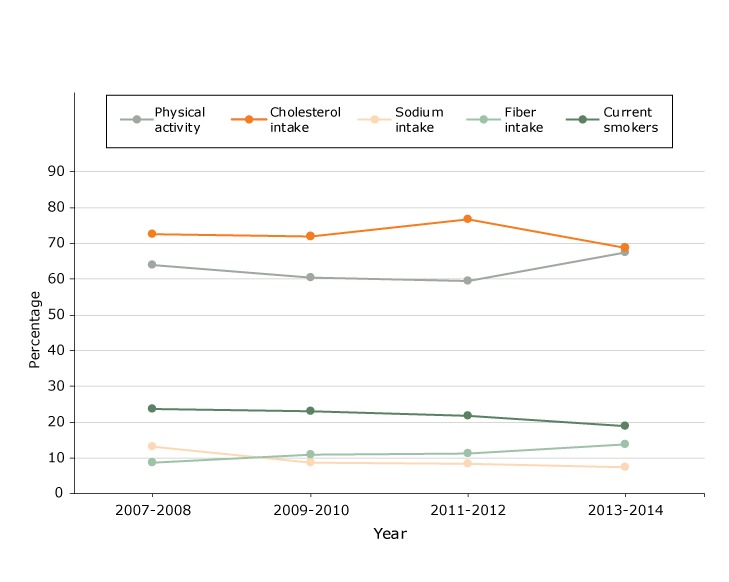
Proportions of women without gestational diabetes (n = 2,747) who met guidelines for health behaviors, National Health and Nutrition Examination Survey, 2007–2014. **Table:** 

In our examination of changes in the proportion of women without gestational diabetes who met guidelines for health behaviors (Figure 2), we found a significant trend only for fiber intake: the proportion increased from 8.7% in 2007–2008 to 13.7% in 2013–2014 (*P* = .04), according to linear regression results. We found no significant trends for the other health behaviors.

Year	Physical Activity, %	Cholesterol Intake, %	Sodium Intake, %	Fiber Intake, %	Current Smokers, %
2007-2008	63.9	72.6	13.2	8.7	23.7
2009-2010	60.4	71.8	8.7	10.9	23.0
2011-2012	59.3	76.8	8.4	11.1	21.9
2013-2014	67.6	68.7	7.4	13.7	18.9

In the multivariate logistic regression analysis, we found no significant difference in practicing the health behaviors between women with gestational diabetes and women without gestational diabetes (Table 2 and Table 3). Some covariates in the models, however, were significant. In the physical activity model, women who self-reported fair or poor health (adjusted odds ratio [AOR] = 0.59; 95% CI, 0.38–0.93) and women who were obese (AOR = 0.63; 95% CI, 0.43–0.92) were less likely to meet the physical activity guideline than were women who self-reported good, very good, or excellent health and women who were not overweight or obese (Table 2).

**Table 2:** Logistic Regression Results of the Association Between Gestational Diabetes Status and Physical Activity and Cigarette Smoking in a Sample of Women Aged 20–44 (n = 3,034), National Health and Nutrition Examination Survey, 2007–2014

Variables	Physical Activity		Currently Smoking	
	Adjusted OR (95% CI)	*P* Value	Adjusted OR (95% CI)	*P* Value
**Gestational diabetes**	1.08 (0.67−1.74)	.74	0.79 (0.52–1.19)	.25
**Year**				
2007–2008	1 [Reference]		1 [Reference]	
2009–2010	0.92 (0.61–1.37)	.66	0.84 (0.61–1.15)	.28
2011–2012	0.82 (0.52–1.28)	.38	0.84 (0.65–1.08)	.18
2013–2014	1.12 (0.69–1.83)	.64	0.59 (0.44–0.78)	<.001
**Age**	1.00 (0.98–1.03)	.70	0.99 (0.91–1.01)	.56
**Race/ethnicity**				
Non-Hispanic white	1 [Reference]		1 [Reference]	
Non-Hispanic black	1.34 (0.88–2.02)	.16	0.54 (0.40–0.73)	<.001
Mexican American	1.15 (0.73–1.80)	.54	0.08 (0.05–0.13)	<.001
Other^a^	1.00 (0.67–1.51)	.99	0.35 (0.24–0.50)	<.001
**Education level**				
<High school graduate	1 [Reference]		1 [Reference]	
High school graduate	0.94 (0.59–1.48)	.78	0.82 (0.60–1.12)	.20
≥Some college	1.10 (0.80 - 1.51)	.54	0.33 (0.25–0.45)	<.01
**Ratio of family income to federal poverty level**				
<100%	1 [Reference]		1 [Reference]	
100%–199%	0.83 (0.54–1.27)	.39	0.66 (0.50–0.89)	<.001
≥200%	1.04 (0.69–1.56)	.85	0.26 (0.18–0.38)	.006
**BMI category**				
Not overweight or obese (<25 kg/m^2^)	1 [Reference]		1 [Reference]	
Overweight (25–30 kg/m^2^)	0.76 (0.52–1.12)	.17	0.90 (0.65–1.24)	.50
Obese (30 kg/m^2^)	0.63 (0.43–0.92)	.02	0.71 (0.52–0.98)	.04
**Health status**				
Good, very good, or excellent	1 [Reference]		1 [Reference]	
Fair or poor	0.59 (0.38–0.93)	.02	1.94 (1.42–2.66)	<.001
**No. of live births delivered**	1.02 (0.88–1.18)	.84	0.97 (0.88–1.07)	.58

Abbreviations: BMI, body mass index; OR, odds ratio.

a Other Hispanic people and other racial groups.

**Table 3:** Logistic Regression Results of the Association Between Gestational Diabetes Status and Dietary Behaviors in a Sample of Women Aged 20–44 (n = 3,034), National Health and Nutrition Examination Survey, 2007–2014

Variables	Cholesterol Intake		Sodium Intake		Fiber Intake	
	Adjusted OR (95% CI)	*P* Value	Adjusted OR (95% CI)	*P* Value	Adjusted OR (95% CI)	*P* Value
**Gestational diabetes**	1.05 (0.75–1.50)	.79	0.75 (0.41–1.37)	.34	0.94 (0.58–1.54)	.81
**Year**						
2007–2008	1 [Reference]		1 [Reference]		1 [Reference]	
2009–2010	0.88 (0.67–1.15)	.33	0.72 (0.50–1.04)	.08	1.38 (0.80–2.38)	.24
2011–2012	1.17 (0.87–1.57)	.28	0.52 (0.32–0.86)	.01	1.72 (0.97–3.06)	.06
2013–2014	0.81 (0.61–1.07)	.13	0.49 (0.31–0.75)	.002	2.03 (1.18–3.49)	.01
**Age**	0.99 (0.98–1.01)	.46	1.02 (0.99–1.05)	.11	1.01 (0.98–1.03)	.59
**Race/ethnicity**						
Non-Hispanic white	1 [Reference]		1 [Reference]		1 [Reference]	
Non-Hispanic black	0.57 (0.44–0.73)	<.001	1.15 (0.83–1.58)	.39	0.54 (0.34–0.87)	.01
Mexican American	0.57 (0.45–0.73)	<.001	0.96 (0.54–1.70)	.88	2.06 (1.44–2.94)	<.001
Other^a^	0.63 (0.48–0.82)	.001	0.76 (0.45–1.29)	.31	1.36 (0.87–2.13)	.18
**Education level**						
<High school graduate	1 [Reference]		1 [Reference]		1 [Reference]	
High school graduate	1.18 (0.86–1.62)	.29	0.99 (0.60–1.64)	.96	0.51 (0.28–0.91)	.02
≥Some college	0.98 (0.75–1.27)	.85	0.77 (0.48–1.24)	.27	1.19 (0.76–1.86)	.45
**Ratio of family income to federal poverty level**						
<100%	1 [Reference]		1 [Reference]			
100%–199%	1.00 (0.77–1.30)	>.99	1.09 (0.74–1.62)	.65	0.99 (0.63–1.57)	.97
≥200%	1.05 (0.79–1.38)	.74	0.44 (0.29–0.69)	<.001	1.36 (0.86–2.13)	.18
**BMI category**						
Not overweight or obese (<25 kg/m^2^)	1 [Reference]		1 [Reference]		1 [Reference]	
Overweight (25–30 kg/m^2^)	0.90 (0.68–1.19)	.44	1.09 (0.68–1.75)	.71	1.08 (0.74–1.58)	.67
Obese (30 kg/m^2^)	0.75 (0.57–0.98)	.03	1.36 (0.89–2.07)	.15	0.85 (0.59–1.22)	.37
**Health status**						
Good, very good, or excellent	1 [Reference]		1 [Reference]		1 [Reference]	
Fair or poor	1.15 (0.91–1.47)	.24	1.19 (0.78–1.81)	.42	0.93 (0.58–1.49)	.77
**No. of live births delivered**	1.03 (0.94–1.12)	.54	1.14 (1.01–1.29)	.04	1.00 (0.87–1.16)	.98

Abbreviations: BMI, body mass index; OR, odds ratio.

a Other Hispanic people and other racial groups.

In the smoking status model, women were less likely (AOR = 0.59; 95% CI, 0.44–0.78) to smoke in 2013–2014 than in 2007–2008. Non-Hispanic black women, Mexican American women, and women in the “other” racial group were less likely to smoke than non-Hispanic white women (*P* < .001). Women with at least some college were less likely (AOR = 0.33; 95% CI, 0.25–0.45) to smoke than those who did not graduate from high school. Women whose annual household income was 100% or more of the federal poverty level were less likely to smoke than women with a lower household income (<100% of the federal poverty level) (*P* < .006). Women who were obese (AOR = 0.71; 95% CI, 0.52–0.98) were less likely to smoke than women who were not overweight or obese, and women who reported fair or poor health (AOR = 1.94; 95% CI, 1.42–2.66) were more likely to smoke than women who reported good, very good, or excellent health (Table 2).

In the cholesterol model, non-Hispanic black women (AOR = 0.57; 95% CI, 0.44–0.73), Mexican American women (AOR = 0.57; 95% CI, 0.45–0.73), women in the “other” racial group (AOR = 0.63; 95% CI, 0.48–0.82), and women who were obese (AOR = 0.75; 95% CI, 0.57–0.98) were less likely to meet the cholesterol intake guideline than the reference groups. In the fiber intake model, non-Hispanic black women (AOR = 0.54; 95% CI, 0.34–0.87) were less likely to meet the fiber intake guideline than non-Hispanic white women, but Mexican American women (AOR = 2.06; 95% CI, 1.44–2.94) were more likely. Women were less likely to meet the sodium intake guideline in 2011–2012 (AOR = 0.52; 95% CI, 0.32–0.86) and in 2013–2014 (AOR = 0.49; 95% CI, 0.31–0.75) than in 2007–2008. Women whose annual household income 200% or more of the federal poverty level were less likely to meet the sodium intake guideline, yet women having more birth deliveries (AOR = 1.14; 95% CI, 1.01–1.29) were more likely to meet this guideline (Table 3).

## Discussion

Our study showed that the prevalence of gestational diabetes is trending upward, but not significantly. The trends for the proportions of women who met guidelines for health behaviors were flat during the study period for women with gestational diabetes and those without. The study period (2007–2014) may have been too short to detect a trend. We found no significant association between gestational diabetes status and practicing the health behaviors examined. Overall, we showed that a substantial number of women with gestational diabetes did not meet healthy behavior guidelines: 35.8% did not meet the guideline for physical activity; 28.2% did not meet the guideline for cholesterol intake; 92.0% did not meet the guideline for sodium intake, 88.7% did not meet the guideline for fiber intake; and 16.5% were current smokers.

Recent studies emphasize the importance of lifestyle interventions and education for women with gestational diabetes (13–15). To prevent the progression of type 2 diabetes, the American College of Obstetrics and Gynecology (16) and the American Diabetes Association (1) recommend that all women at high risk for diabetes receive healthy lifestyle education on diet, physical activity, and weight management. The Diabetes Prevention Program showed that lifestyle interventions reduced type 2 diabetes incidence by 35% in women with gestational diabetes (17).

Our study showed that during 2007–2014, 8.9% of women of reproductive age reported a history of gestational diabetes, which aligns with previous estimates of gestational diabetes prevalence (18,19). The 2007–2010 Pregnancy Risk Assessment Monitoring System, for example, showed that 9.2% of women delivering live births had a diagnosis of gestational diabetes (20).

Although the 2007–2014 NHANES data did not show a significant increasing trend in gestational diabetes prevalence, previous studies, mostly using inpatient data sets, did show such a trend. For example, one study of National Hospital Discharge Survey data reported that the rate of gestational diabetes increased significantly among females aged 15 to 49, from 0.3% in 1979–1980 to 5.8% in 2008–2010 (7). Another study analyzing data from the Agency for Healthcare Research and Quality’s State Inpatient Databases found that the prevalence of gestational diabetes increased from 3.71 per 100 deliveries in 2000 to 5.77 per 100 deliveries in 2010 (8). The lack of a significant trend in gestational diabetes prevalence in our study may be due to the shorter time span — only 4 waves of data were available for our analysis. The trend should continue to be monitored.

Our results showed that women with gestational diabetes were not more likely or less likely to meet the guidelines for health behaviors than women without gestational diabetes. We expected that women with a history of gestational diabetes would have higher levels of healthy behaviors. Our findings are consistent with a 2006 study on health behaviors in women of childbearing age (5), which found no difference in physical activity and other health behaviors between women with and without gestational diabetes. Our study results suggest that more education efforts are needed to promote healthy behavior practices among these women.

Some studies found that women with gestational diabetes, citing concern for the health of the baby, did report making healthy behavior changes during pregnancy. After the birth, however, women reported obstacles, such as fatigue, lack of time, and lack of child support, to practicing healthy behaviors (21,22). Additionally, women with gestational diabetes typically are closely monitored by a team of medical providers during pregnancy. After pregnancy, healthy behaviors may be hard to sustain because of fragmentation of care (23). One study found that routine glucose tolerance testing after a gestational diabetes pregnancy was not practiced by physicians (24). Future research should investigate interventions to remove physician barriers to promoting women’s health after pregnancy.

We found some racial differences in practicing health behaviors. Women in racial/ethnic minority groups were less likely to be current smokers and to meet cholesterol intake guidelines than non-Hispanic white women, and Mexican American women were more likely than non-Hispanic white women to meet fiber guidelines. One qualitative study found that Vietnamese women were most likely to follow diet and exercise plans while white women were least likely (25). Thus, understanding how various racial/ethnic groups interpret health messages may be important, and health educators may need to tailor their messages for these groups. Furthermore, we showed that a higher income level did not necessarily increase the odds of practicing healthy behaviors. For example, higher-income women were not more likely than low-income women to meet the sodium intake guideline. This finding is consistent with data from the Interdisciplinary Chronic Disease Collaboration survey, which found no significant difference in following behavioral change advice among various income groups (26). Thus, factors other than income may play a more important role in behavior change among reproductive-aged women. Higher income women were, however, more likely to be nonsmokers.

Our study found some differences over time in dietary behaviors and smoking. Women in 2013–2014 were less likely than women in 2007–2008 to meet the daily sodium intake guideline, which may reflect consumption of high-sodium fast food. A study found that the sodium content in 8 leading US fast-food restaurants increased by 23.4% from 1997–1998 to 2009–2010 (27). Studies also found that women aged 20 to 39 were more likely to consume fast food than women in other age groups (28). On a positive side, our study found that women were less likely to smoke in 2013–2014 than in 2007–2008 and were more likely to meet the fiber intake guideline in 2011–2012 and 2013–2014 than in 2007–2008. The decrease in smoking rate may be related to increases in state cigarette taxes (29). For fiber intake, changes in SNAP (Supplemental Nutrition Assistance Program) and WIC (Special Supplemental Nutrition Program for Women, Infants, and Children) may be a factor. A revision of the SNAP for WIC program in 2009 increased the availability and accessibility of high-fiber produce in WIC-certified vendors (30). These reforms allow mothers to more easily shop for healthy foods.

This study has limitations. First, the data are self-reported, and such data are subject to bias. Respondents may misreport or underreport gestational diabetes and may overreport healthy behaviors, thus affecting the accuracy of the estimates. Second, the short study period — only 4 waves of data — may not be sufficient for trend analysis. Our study provided an initial assessment of the changes from 2007 through 2014. Future research is needed to assess the trend when more data are available. Third, we did not include such factors as employment status in our model, which could have affected the estimates of the association between gestational diabetes status and health behaviors. We did not include the number of children in the home because these data were not available in 2007–2009 NHANES. Contextual variables were not included in the model because these data are not available in NHANES. Future study should investigate environmental barriers to adopting healthy life behaviors.

NHANES data showed that the prevalence of gestational diabetes did not change and the practice of healthy behaviors did not increase significantly from 2007–2008 to 2013–2014. Many women with gestational diabetes did not meet guidelines for healthy behaviors. Given the high risk of type 2 diabetes, practicing healthy behaviors is essential to preventing type 2 diabetes. Barriers to healthy behavior involve intrapersonal and interpersonal factors as well as system-level factors. Thus, coordinated intervention programs are needed to promote and assist women with a history of gestational diabetes in adopting health behaviors.
